# Cardiac Magnetic Resonance Imaging in the Evaluation and Prognosis of Infiltrative Cardiomyopathies

**DOI:** 10.3390/jcdd12040154

**Published:** 2025-04-12

**Authors:** Merna Hussien, Francisca Bermudez, Patrick T. Bering, Gaby Weissman, Allison G. Hays, Farooq H. Sheikh

**Affiliations:** 1 Department of Cardiology, MedStar Washington Hospital Center, Washington, DC 20010, USA; 2 School of Medicine, Georgetown University, Washington, DC 20007, USA; 3 Department of Cardiology, School of Medicine, Johns Hopkins University, Baltimore, MD 21205, USA

**Keywords:** cardiac MRI, infiltrative cardiomyopathy, amyloidosis, sarcoidosis, hemochromatosis, iron overload cardiomyopathy, Fabry

## Abstract

Advancements in cardiac magnetic resonance (CMR) imaging quality and availability have made it an essential tool in the care of individuals living with cardiomyopathies. CMR complements clinical suspicion, electrocardiogram patterns, and echocardiographic findings to help elucidate the etiology of cardiomyopathies and can also be used to prognosticate and follow treatment responses. In this review, we highlight the common CMR findings in cardiac amyloidosis, cardiac sarcoidosis, iron overload cardiomyopathy, and Fabry disease. We also summarize prognostic findings and additional potential roles for CMR in the management of infiltrative cardiomyopathies.

## 1. Introduction

**Figure 1:**
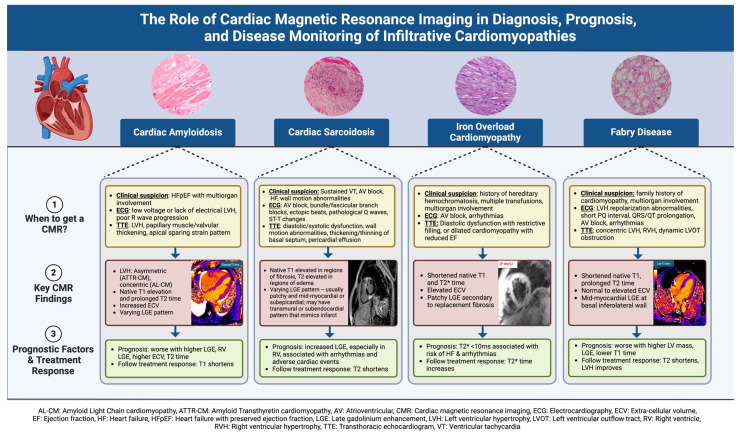
Summary of clinical presentation, key CMR findings, prognostic factors of CMR findings, and role of CMR in following treatment response in cardiac amyloidosis, cardiac sarcoidosis, iron overload cardiomyopathy, and Fabry disease.

Infiltrative cardiomyopathies are a heterogeneous group of conditions that affect the heart muscle, resulting in heart failure and other cardiac diseases. They encompass a wide spectrum of pathophysiology, ranging from infiltration of the extracellular space with misfolded proteins in cardiac amyloidosis, granulomatous inflammation in cardiac sarcoidosis, intracellular deposition of iron in primary and secondary hemochromatosis, to glycogen storage disorders such as Fabry disease [1]. Infiltrative cardiomyopathies have been considered a rare form of heart muscle disease, remaining underdiagnosed and frequently mistaken for hypertensive cardiomyopathy [2,3]. Given the available therapies for many infiltrative diseases, there is value in early diagnosis in hopes of halting disease progression. Clinical history, electrocardiography (ECG), and echocardiography provide helpful clues towards diagnosing infiltrative cardiomyopathies, but advanced imaging such as cardiac magnetic resonance imaging (CMR) helps to provide clues for an early diagnosis and may provide prognostic value. We conducted a narrative review to summarize the common CMR characteristics and prognostic markers in some infiltrative cardiomyopathies, including cardiac amyloidosis, cardiac sarcoidosis, hemochromatosis, and Fabry disease (Figure 1). The narrative review allowed for a flexible exploration of this topic to provide pertinent information to clinicians taking care of patients with stated cardiomyopathies.

## 2. Basics of Cardiac Magnetic Resonance Imaging

The CMR is a powerful noninvasive imaging tool able to evaluate myocardial tissue structure and function with high spatial resolution and lack of radiation exposure. By utilizing different sequences of radiofrequency pulses to affect the magnetization and relaxation time of protons in tissue, distinct signal intensities that reflect tissue properties are produced [4]. The most common CMR contrast agent used to help characterize abnormalities in the myocardium is chelated with gadolinium (Gd). Gd is a water-soluble heavy metal that diffuses freely between the intravascular and interstitial space outside the brain but is unable to cross to the intracellular space [5]. Therefore, scarred or infarcted myocardial tissue holds a higher concentration of Gd due to loss of intact myocytes and increased intracellular space. Gd affects the magnetic dynamics of tissue and specifically shortens the longitudinal relaxation time (T1) of protons and, therefore, appears as enhanced (bright) on T1-weighted images [5]. It is important to note that increased signal or late gadolinium enhancement (LGE) can reflect interstitial fibrosis, which can be diffuse in the myocardium, replacement fibrosis, which tends to be brighter and more focal, or areas of inflammation. By combining the evaluation of ventricular structure, function, and pattern of LGE, infiltrative processes can be identified on CMR and differentiated from acute or chronic myocardial infarction based on different tissue characteristics. Accordingly, recent AHA/ACC/HFSA and ESC guidelines give a class 2a recommendation to obtain CMR in patients with new heart failure or cardiomyopathy [6,7].

## 3. Cardiac Amyloidosis

### 3.1. Pathophysiology and Clinical Presentation

Amyloidosis is a broad spectrum of systemic diseases caused by the aggregation of proteins misfolded into insoluble fibrils in the extracellular space. The most common forms of amyloidosis that involve the heart are monoclonal amyloid light chain cardiomyopathy (AL-CM) and amyloid transthyretin cardiomyopathy (ATTR-CM)-variant and wild type [2]. Cardiac involvement typically results in restrictive cardiomyopathy but can also cause abnormal conduction, coronary, and pericardial disease. Suspicion for cardiac amyloidosis should be high if a patient is presenting with concomitant signs of multiorgan involvement, such as proteinuria, neuropathy, or gastrointestinal dysmotility and malabsorption. The 2023 ACC expert consensus decision pathway highlighted specific extracardiac red flags, including bilateral carpal tunnel syndrome, spontaneous biceps tendon rupture, autonomic dysfunction, and gastroparesis [8].

### 3.2. ECG Findings

ECG hallmark findings reflect the displacement of myocytes with amyloid fibrils resulting in low voltage QRS or lack of electrical left ventricular hypertrophy (LVH), poor R-wave progression, and, less commonly, atrioventricular (AV) node block. Atrial fibrillation is a relatively common arrhythmia in cardiac amyloidosis, given restrictive left ventricular (LV) filling and resultant atrial myopathy [9].

### 3.3. Echocardiography Findings

On echocardiography, LV wall thickening with preserved or reduced LV size, bi-atrial enlargement, and preserved LV ejection fraction should raise suspicion for amyloid CM [10]. Valvular and papillary muscle thickening can also occur, along with pericardial or pleural effusions. Diastolic dysfunction of higher grade showing restrictive pattern can favor amyloid CM versus hypertensive LVH [11]. While LV ejection fraction remains preserved until end-stage amyloid CM, left ventricular speckle-tracking-derived longitudinal strain can show preceding global and regional deformation of the myocardium with added diagnostic value [12]. An apical sparing strain pattern, in which the LV apical segments have normal or supranormal strain and the basal segments have reduced strain, can have diagnostic value in amyloid CM. An early study showed that an apical sparing pattern ratio ≥ 1, calculated by dividing the average apical longitudinal strain by the average mid and basal-longitudinal strain, had high diagnostic accuracy (93% sensitivity, 82% specificity) in discriminating patients with known cardiac amyloidosis compared to controls [12]. Another study that included AL-CM and ATTR-CM demonstrated an apical sparing strain pattern in 48% of patients [13]. However, in a larger study of 1187 patients referred to amyloid centers in Europe, the accuracy of apical sparing for detecting amyloid CM was less than in earlier reports, with a sensitivity of 58% in patients with systemic AL amyloidosis [14]. Therefore, while an apical sparing pattern can be seen in amyloid CM and may increase the likelihood of diagnosis, its absence should not eliminate the possibility of amyloid CM and further workups that may be necessary [15]. Global longitudinal strain (GLS) can be used to derive the global left ventricular myocardial work, which has been shown to overcome the loading-dependent limitation of GLS and to be a better discriminator of amyloid CA from hypertrophic cardiomyopathy (HCM) or hypertensive controls over GLS alone [16]. In addition to left ventricular strain analysis, speckle-tracking echocardiography can be used to analyze left atrial strain, which was found to be significantly impaired in amyloid CM when compared to HCM and control groups [17]. This impairment was persistent irrespective of the left ventricular EF in amyloid CM.

### 3.4. CMR Findings

CMR can help differentiate amyloid CM from other infiltrative or hypertrophic cardiomyopathies. It is a complementary diagnostic tool to serological workup, bone scintigraphy, and endomyocardial biopsy. CMR can also offer prognostic and disease-monitoring ability. Cine steady-state free precession (SSFP) sequences examine the morphology and function of the four cardiac chambers. Like echocardiography, SSFP in amyloid CM shows increased LV wall thickness, atrial septal thickness, and valvular thickness with normal or reduced biventricular function. LV septal hypertrophy is mostly asymmetric in ATTR-CM and concentric in the majority of AL-CM [18].

Amyloid deposition affects myocardial tissue architecture, which results in elevated intrinsic, non-contrasted myocardial T1 signal (native T1 mapping) [4]. The progression of amyloid CM can be quantitively monitored with native T1 elevation, which correlates with the degree of amyloid infiltration [19]. Amyloid fibril deposition can also cause myocardial edema. The increased water content in tissue prolongs T2 time, which is the relaxation time of protons in the transverse plane. However, T2 time in amyloid CM is usually shorter than that reported in myocarditis or myocardial infarction [20].

Post-contrast CMR imaging provides information about the extracellular space, which in amyloid CM is distorted by amyloid deposition. In amyloid CM, TI-scout images for gadolinium kinetics show a simultaneous nulling of the blood pool and myocardium or a reverse null pattern where the myocardium nulls prior to the blood pool [21]. LGE patterns in amyloid CM range from absence to subendocardial or transmural, correlating with the severity of infiltration. LGE often involves all cardiac chambers. Contrast-enhanced T1-weighted imaging can be used to estimate extracellular volume (ECV), which is often markedly increased in amyloid CM (>40%) due to extracellular amyloid deposition [22]. Elevated ECV was shown to precede LGE in certain cases with a high probability of amyloid CM, likely signaling early disease [23]. However, the use of Gd to evaluate LGE and ECV may be precluded in patients with advanced kidney disease. Other contrast-free analyses of CMR that can help distinguish amyloid CM include longitudinal strain analysis. Similar to echocardiography, CMR shows globally reduced longitudinal strain in amyloid CM with relative apical sparing with specificity to distinguish CA of 82% but sensitivity of only 43% [24]. If Gd can be administered, the LGE ratio between base and apex, in addition to the relative strain ratio, was shown to have a higher discriminatory ability to distinguish amyloid CM from other causes of LVH [24]. Key CMR findings in amyloid CM are summarized in Table 1.

### 3.5. The Role of CMR in Prognosis and Disease Monitoring

Many of the above-mentioned parameters were shown to have prognostic value in both AL-CM and ATTR-CM, namely the higher amount of LGE, presence of right ventricular (RV) LGE, and increased size of ECV [18,25,26,27]. T2-mapping was also shown to be an independent predictor of mortality in AL-CM [20]. T1 mapping and ECV have been shown to be disease burden markers that can be monitored throughout treatment, which is likely due to their ability to estimate amyloid fibril deposition burden [18,28].

## 4. Fabry Disease

### 4.1. Pathophysiology and Clinical Presentation

Fabry disease (also known as Anderson–Fabry disease) is a rare X-linked lysosomal storage disorder. It is caused by loss-of-function mutations in the alpha-galactosidase A enzyme (alpha-Gal A) gene. With a deficient alpha-Gal A enzyme, globotriaosylceramide (Gb3) accumulates in lysosomes of different body cells, resulting in cytotoxic and proinflammatory effects [29]. The phenotype of Fabry disease (FD) varies widely depending on the residual enzyme activity. Heterozygous females usually have a milder and later presentation, but due to skewed X-chromosome inactivation, can sometimes have a severe presentation. Individuals with classic FD have very low to no alpha-Gal A enzyme activity. They usually present with early neurologic symptoms, and also develop dermatologic, gastrointestinal, renal, and cardiac manifestations, with cardiac manifestations appearing later, in the third to fourth decade of life [30]. Late-onset cardiac variants of FD have also been reported and can be mistaken for idiopathic HCM [31,32]. In fact, the prevalence of FD in patients with late-onset HCM is 6% in men and 12% in women in two cohort studies [33,34].

### 4.2. ECG Findings

Cardiac involvement is the main driver of mortality in individuals with FD [35]. FD cardiomyopathy encompasses progressive LVH, myocardial fibrosis, diastolic dysfunction, inflammation, and arrhythmias [36]. ECG often shows evidence of LVH with repolarization abnormalities, short PQ interval, and QRS/QT prolongation. AV block, bradyarrhythmias, and atrial/ventricular tachyarrhythmias can also occur [29].

### 4.3. Echocardiography Findings

On echocardiography, concentric LVH and prominent papillary muscles are common findings of FD cardiomyopathy. LVH can be accompanied by systolic anterior motion of the mitral valve and left ventricular outflow tract (LVOT) obstruction, especially after exertion [37]. Right ventricular hypertrophy was also reported in varying prevalence in FD cardiomyopathy between 40% and 70%, along with preserved RV systolic function [38]. Spectral strain imaging offers further value in detecting early LV dysfunction independent of LVH and preceding LVEF reduction, which develops in later stages. For instance, the global longitudinal strain (A) of the LV can be reduced in early FD cardiomyopathy with a prevalent involvement of the inferolateral wall. The global circumferential strain analysis shows a loss of the base-to-apex circumferential strain gradient, which is usually preserved in HCM [39].

Given the rarity of the disease and the heterogeneity of presentation, high clinical suspicion is needed to refer patients to diagnostic testing. Early diagnosis of FD is crucial as timely treatment with enzyme replacement or chaperone therapy can prevent progression to severe organ failure. The diagnosis is reliably made by biochemical assays testing alpha-Gal A enzyme activity and/or genetic testing [29,40]. FD cardiomyopathy should be considered in individuals with unexplained LVH.

### 4.4. CMR Findings

CMR plays a multifaceted role in FD cardiomyopathy. It supports diagnosing and differentiating FD cardiomyopathy from other causes of LVH. With higher spatial resolution and the ability to detect patterns of cardiac fibrosis, it can reveal early cardiac involvement of the disease, and, hence, enable early treatment. It also provides prognostic value and accurate monitoring of disease progression [41].

On CMR SSFP, better quantification of LV and papillary muscle mass can be made and tracked over time compared to echocardiography. Glycosphingolipid accumulation in myocytes results in a shorter T1 relaxation time on the T1 mapping sequence. In a systemic review, the mean T1 value in FD patients was 935 +/− 48 ms with a 1.5 T CMR scanner, compared to 999 + 31 ms in controls [42]. Shortened native T1 time has been described in individuals with FD without LVH and correlates with a reduced GLS on echocardiography [43]. Pseudo-normalization of native T1 time can be noted in areas of myocardial fibrosis or inflammation [44]. Native T2 mapping can show elevated T2 relaxation time in the basal inferolateral region or other areas of LGE, which reflects myocardial inflammation [41]. Post-contrast imaging would classically show a pattern of LGE involving the mid-myocardium of the basal inferolateral wall. However, LGE may become more extensive in advanced cases [45]. ECV values are normal in FD but may increase in areas of fibrosis. Key CMR findings in Fabry disease are summarized in Table 2.

### 4.5. The Role of CMR in Prognosis and Disease Monitoring

CMR can risk stratify patients with FD cardiomyopathy and provide prognostic value. For instance, higher LV mass index and more extensive LGE are strongly correlated with the incidence of cardiovascular events, including heart failure, arrhythmias, and cardiac death [46]. Lower native T1 time is also prognostic as it reflects Gb3 accumulation. Orsborne et al. included age, LV mass index, and native T1 time in a prognostic model that estimates the 5-year risk of adverse cardiovascular events [47].

CMR is used to monitor the response to therapy for FD. The response of LVH to therapy has been shown to be variable, with patients with baseline LVH and minimal LGE having more reduction [48]. T2 time reduces with successful treatment. T1 time would be expected to normalize with therapy as well, however, there have not been studies demonstrating the utility of monitoring T1 time with therapy [40]. Expert groups suggest routinely obtaining CMR every 2–5 years before the onset of cardiac features and then every 2–3 years in patients with progressive disease to monitor the progression of FD cardiomyopathy and evaluate the success of therapy [49,50,51].

## 5. Iron Overload Cardiomyopathy

### 5.1. Pathophysiology and Clinical Presentation

Iron overload can occur as a primary condition of increased intestinal absorption, such as in hereditary hemochromatosis, or as an acquired condition secondary to increased oral or parenteral intake, such as in transfusion-dependent anemias. When transferrin is oversaturated with iron, unbound iron causes oxidative stress to cells, eventually leading to their apoptosis. Iron overload, therefore, can result in multiorgan dysfunction involving the liver, skin, endocrine system, neurological system, and heart [52]. Cardiac involvement tends to occur later than hepatic involvement and is a major driver of morbidity and mortality in iron overload conditions [53,54]. In the heart, iron first accumulates in the ventricles, followed by the atria, in an epicardial to endocardial progression pattern. In turn, it can result in diastolic, tachy- or brady-arrhythmias, and eventually systolic dysfunction and pulmonary hypertension [53,54,55]. Iron overload cardiomyopathy (IOC) can, therefore, have a range of presentations depending on the extent of iron deposition. Extracardiac clues to iron overload include cirrhosis, endocrine abnormalities like diabetes mellitus, hypothyroidism, and hypogonadism, and skin hyperpigmentation [56]. Initial diagnostic evaluation with elevated ferritin (>200 ng/mL for women and >300 ng/mL for men) and transferrin saturation (55% or above) marks the diagnosis of systemic iron overload, but further testing is needed to evaluate for IOC [57].

### 5.2. ECG Findings

ECG can be nondiagnostic in early IOC. As iron deposition increases and involves the atria, supraventricular tachycardias, including atrial fibrillation, can be seen. Ventricular arrhythmias can be seen with reduced LV EF. Prolonged PR and higher degree atrioventricular blocks can occur when the conduction system is affected by iron depositions [58,59].

### 5.3. Echocardiography Findings

Although echocardiography cannot directly evaluate iron deposition, it serves as an initial imaging modality to evaluate the cardiac structure and function in IOC. Initially, diastolic dysfunction as evaluated by conventional parameters, including mitral inflow velocities (E/A ratio), tissue Doppler velocities (e′, and E/e′), and ventricular GLS, and atrial strain can be seen in asymptotic individuals with proven cardiac iron deposition [60,61,62,63]. In later disease stages, the RV and LV may dilate and have reduced systolic function, and pulmonary hypertension signs may be seen with either phenotype of LV dysfunction (diastolic with restrictive filling vs. dilated with reduced systolic function) [56,64].

### 5.4. CMR Findings

In addition to providing an accurate structural and functional assessment of cardiac chambers in IOC, CMR can quantify cardiac iron overload. Stored iron molecules in the form of hemosiderin act as small magnets when placed in a strong magnetic field, which shortens the relaxation time of tissue protons by disrupting their coherence. This can be measured more specifically as a T2*, which is the half-life time of signal intensity decay obtained using a gradient echo rather than a spin echo in T2 [65]. T2* is inversely proportional to the tissue iron contact. T2* measured within the interventricular septum is considered highly representative of global myocardial iron and has prognostic implications, as discussed below [65,66,67].

T1 mapping is complementary to T2* and was shown to be concordant [68]. Native T1 time will be shortened with iron overload. Studies suggest that while T1 time may be more sensitive for cardiac iron content, it is less specific as it can be affected by myocardial fibrosis and fat [68,69]. Patchy areas of LGE can be seen secondary to replacement fibrosis [70]. In individuals with β-thalassemia major and myocardial iron overload, ECV was found to be increased and associated with a history of heart failure [71]. Key CMR findings in IOC are summarized in Table 3.

### 5.5. The Role of CMR in Prognosis and Disease Monitoring

CMR can identify preclinical iron overload and enable early initiation of iron-chelation therapy. T2* can be used to risk stratify patients with IOC. T2* less than 20 ms is considered the threshold for myocardial iron overload. Values less than 20 ms were found to correlate with a reduction in LVEF, and values less than 10 ms were found to be associated with a higher risk of developing heart failure and arrhythmias in prospective studies of individuals with β-thalassemia major [67,72,73,74]. T2* value, therefore, helps guide the intensity of chelation therapy and is followed up to ensure improvement and response to therapy [56].

## 6. Cardiac Sarcoidosis

### 6.1. Pathophysiology and Clinical Presentation

Sarcoidosis is a systemic inflammatory disease characterized by the formation of noncaseating granulomas in almost any organ [75]. It is believed that sarcoidosis occurs secondary to the interplay of genetic predisposition, environmental exposure, and immunological dysregulation [76]. It affects individuals from all races and ethnicities, with a higher prevalence in African Americans and those of Scandinavian origin [77,78]. It also affects people of all ages, but incidence peaks at 30 to 50 years in men and 50 to 60 years in women, with a slightly higher prevalence in women [79].

While the lungs are the most affected organs, cardiac involvement is a significant driver of morbidity and mortality in sarcoidosis [76]. Cardiac sarcoidosis (CS) with overt clinical symptoms was found to affect 5% of those with sarcoidosis, but autopsy data showed clinically silent cardiac involvement in up to 25% of patients with sarcoidosis [80,81,82]. Manifestation of CS can range from subclinical cardiomyopathy to arrhythmias and heart failure. A high clinical suspicion for CS should be raised by unexplained sustained ventricular tachycardia, atrioventricular blocks in young individuals, unexplained wall motion abnormalities or aneurysms, and unexplained cardiomyopathy in individuals without a history of extracardiac sarcoidosis [83]. Screening for CS in individuals with known extracardiac sarcoidosis should be prompted by symptoms such as palpitations or syncope, ECG abnormalities, or cardiac imaging abnormalities [83].

Making a definitive diagnosis of CS requires histological evidence of sarcoidosis in the heart, and with the low diagnostic yield of endomyocardial biopsy for CS, multimodality imaging such as echocardiography, CMR, and ^18^F-fluorodeoxyglucose positron emission tomography (FDG-PET) play a supportive diagnostic role emphasized in the several diagnostic criteria for CS [84,85,86].

### 6.2. ECG Findings

The ECG is usually normal in asymptomatic CS [82]. ECG abnormalities in symptomatic CS include atrioventricular blocks, bundle and fascicular branch blocks, ectopic beats, pathological Q waves, and ST-T changes [82].

### 6.3. Echocardiography Findings

In clinically silent CS, only GLS may be abnormal and is associated with future occurrence of cardiac events [87]. As clinical manifestations arise, the echocardiogram abnormalities in CS include diastolic dysfunction, systolic dysfunction, wall motion abnormalities not following a coronary distribution, wall thickening or thinning, especially at the basal septum, and pericardial effusion [88,89,90].

### 6.4. CMR Findings

CMR is a sensitive diagnostic tool for CS, with studies yielding sensitivities between 75 and 100% [88]. SSFP cine imaging is used to assess for regional wall thinning or thickening, aneurysmal or dyskinetic segments, and biventricular systolic function [91]. In CS, native T1 mapping may be focally increased in regions of fibrosis or edema [92]. Native T2 mapping may also be focally increased in regions of edema if there is active inflammation [92]. Black-blood T2 weighted imaging can improve the detection of myocardial edema and was found to have comparable diagnostic performance to FDG-PET in a retrospective study [93].

The hallmark of CMR in CS is the evaluation of LGE. LGE can reflect edema in the acute inflammatory phase and replacement fibrosis in the chronic stage of CS. LGE is often patchy, mid-myocardial and/or subepicardial, and involves the basal LV segments and RV portion of the septum [88,94]. Sometimes, the LGE may be transmural or subendocardial, mimicking an ischemic pattern [95]. FDG-PET imaging complements CMR, and combining both modalities has been shown to enhance the certainty of CS diagnosis [96]. In a meta-analysis of histologically proven cardiac sarcoidosis, Okasha et al. found that the lack of any myocardial LGE or the presence of isolated midmyocardial, subendocardial, or transmural LGE were rarely to never present [97]. This highlights the potential role of certain LGE patterns on CMR in excluding cardiac involvement in sarcoidosis. Key CMR findings in CS are summarized in Table 4.

### 6.5. The Role of CMR in Prognosis and Disease Monitoring

In addition to its diagnostic sensitivity, CMR offers prognostic value in CS. The presence of LGE, irrespective of LVEF, was found to correlate with arrhythmic risk and major adverse cardiac events [98,99]. Studies have looked at whether certain patterns of LGE were associated with a higher risk for arrhythmia and mortality. In a recent meta-analysis including 899 patients, those with right ventricular LGE had a significantly higher risk of composite events (all-cause death, cardiovascular events, or sudden cardiac death) (RR: 4.8 [95% CI: 2.4–9.6]; *p* < 0.01) and a higher risk for sudden cardiac death (RR: 9.5 [95% CI: 4.4–20.5]; *p* < 0.01) [100]. Therefore, the presence of LGE on CMR, especially right ventricular LGE, despite an LVEF greater than 35%, could prompt a referral for a primary prevention implantable cardioverter–defibrillator.

## 7. Conclusions

**Figure 2:**
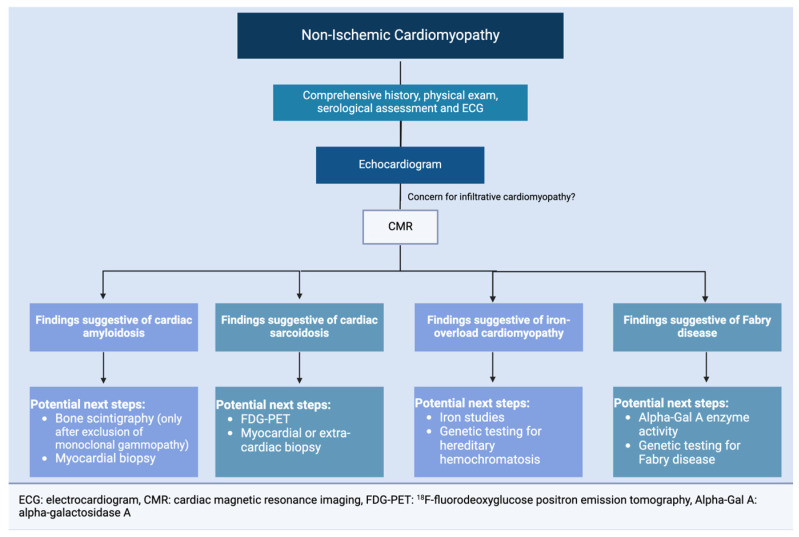
Proposed diagnostic algorithm for the use of CMR when there is clinical concern for infiltrative cardiomyopathies, as summarized in Figure 1.

CMR plays a pivotal role in the diagnosis, prognosis, and disease monitoring of various infiltrative cardiomyopathies. It complements information from other imaging modalities and offers a unique assessment of the myocardium and other cardiac structures. It is becoming a cornerstone in evaluating nonischemic cardiomyopathy, especially when an infiltrative process is suspected (Figure 2). Improving access and availability of CMR is paramount for the care of patients living with cardiomyopathy.

## Figures and Tables

**Table 1:** Key CMR findings in amyloid cardiomyopathy.

CMR Parameter	Key CMR Findings	Illustrative Example
Cardiac Structure and Function	Increased left ventricular wall thickness: o Asymmetric in ATTR-CM o Concentric in AL-CM Increased valvular thickness Normal or reduced biventricular systolic function Pericardial Effusion	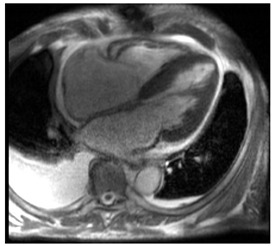
T1 time	Elevated native T1 time	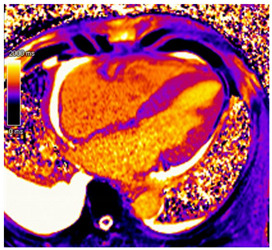
T2 Time	Possibly elevated T2 time but not as high as reported in myocarditis or myocardial infarction	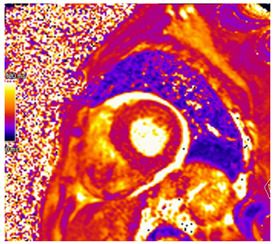
LGE	Diffuse patchy LGE of the left ventricle LGE of the atria and the right ventricle may be present	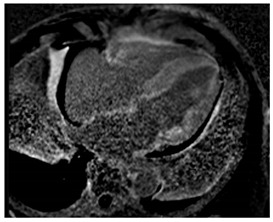
ECV	Markedly elevated ECV	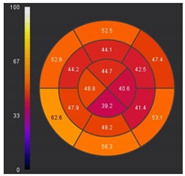

**Table 2:** Key CMR findings in Fabry disease.

CMR Parameter	Key CMR Findings	Illustrative Example
Cardiac Structure and Function	Increased left ventricular mass with more advanced disease Reduced left ventricular systolic function with more advanced disease	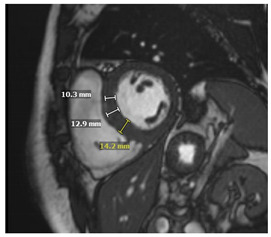
T1 time	Short native T1 time	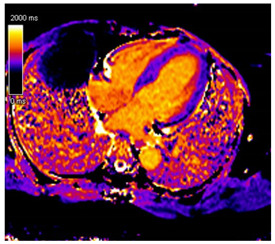
T2 Time	Possibly elevated T2 time in the basal inferolateral wall or in areas of LGE	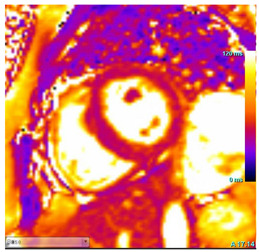
LGE	Mid-myocardium LGE of the basal inferolateral wall	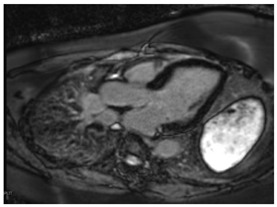

**Table 3:** Key CMR findings in iron overload cardiomyopathy.

CMR Parameter	Key CMR Findings	Illustrative Example
Cardiac Structure and Function	Dilated ventricular chambers with reduced systolic function in later stages	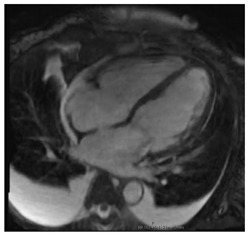
T1 time	Short native T1 time	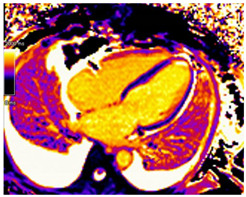
T2 Time	Short T2 time	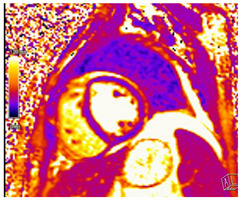
T2* Time	Short T2* time, which inversely correlates with myocardial iron content T2* time less than 20 ms is the threshold of myocardial iron overload	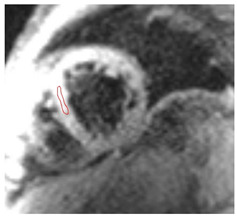
LGE	Patchy areas of LGE can be seen secondary to replacement fibrosis	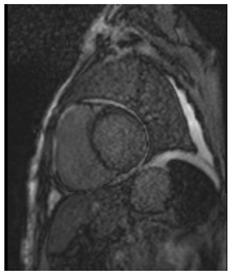

**Table 4:** Key CMR findings in cardiac sarcoidosis.

CMR Parameter	Key CMR Findings	Illustrative Example
Cardiac Structure and Function	Regional wall thinning or thickening Aneurysmal or dyskinetic segments Biventricular systolic function in more severe cases	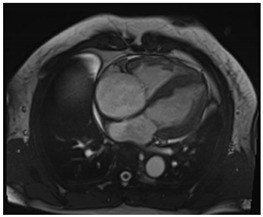
T1 time	Native T1 time may be focally increased in regions of fibrosis or edema	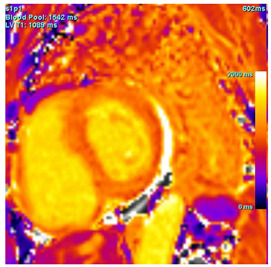
T2 Time	T2 time may be focally increased in regions of edema	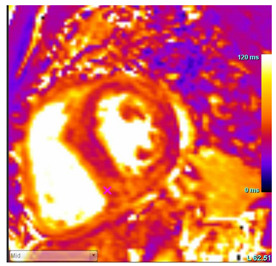
LGE	Patchy, mid-myocardial and/or subepicardial and involves the basal LV segments and RV portion of the septum LGE may be transmural or subendocardial, mimicking an ischemic pattern	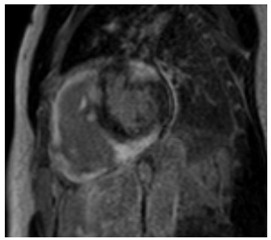

## Data Availability

No new data were created or analyzed in this study. Data sharing is not applicable to this article.

## Abbreviations

The following abbreviations are used in this manuscript:

ACC	American College of Cardiology
AHA	American Heart Association
AL-CM	Amyloid Light Chain Cardiomyopathy
alpha-Gal A	alpha-galactosidase A enzyme
ATTR-CM	Transthyretin-derived Cardiomyopathy
AV	Atrioventricular
CM	Cardiomyopathy
CMR	Cardiac Magnetic Resonance
CS	Cardiac Sarcoidosis
ECG	Electrocardiography
ECV	Extra-cellular volume
EF	Ejection fraction
ESC	European Society of Cardiology
FD	Fabry Disease
FDG-PET	18F-fluorodeoxyglucose positron emission tomography
Gb3	Globotriaosylceramide
Gd	Gadolinium
GLS	Global longitudinal strain
HCM	Hypertrophic cardiomyopathy
HFSA	Heart Failure Society of America
IOC	Iron overload cardiomyopathy
LGE	Late gadolinium enhancement
LV	Left ventricle
LVH	Left ventricular hypertrophy
LVOT	Left ventricular outflow tract
RV	Right Ventricle
SSFP	Steady-State Free Precession
